# Characterization of User-Centered Security in Telehealth Services

**DOI:** 10.3390/ijerph16050693

**Published:** 2019-02-26

**Authors:** Mario Vega-Barbas, Fernando Seoane, Iván Pau

**Affiliations:** 1 Institute of Environmental Medicine, Karolinska Institutet, 171 77 Stockholm, Sweden; 2 Institute for Clinical Science, Intervention and Technology, Karolinska Institutet, 141 57 Huddinge, Sweden; fernando.seoane@ki.se; 3 Department of Biomedical Engineering, Karolinska University Hospital, 14157 Huddinge, Sweden; 4 Swedish School of Textiles, University of Borås, 501 90 Borås, Sweden; 5 Intelligent Spaces and Services Research Group, Universidad Politécnica de Madrid, 28031 Madrid, Spain; ivan.pau@upm.es

**Keywords:** telehealth, technology acceptance, sensitive services, usability, personalization, user-centered Security

## Abstract

Emerging information and communication technologies are expected to foster new, efficient and accessible services for citizens, while guaranteeing the core principles of equality and privacy. Telehealth services are a clear example of a service in which technology can help enhance efficiency. The security of telehealth services is essential due to their critical nature. However, although ample efforts have been made to characterize security requirements for healthcare facilities, users are often worried because they are not aware of or do not understand the guarantees provided by the technology they are making use of. This paper describes the concept of User-Centered Security and characterizes it in the form of requirements. These requirements have been formalized in the form of a security architecture that should be utilized for each telehealth service during its design stage. Thus, such sensitive services will adequately manage patient fears regarding their correct operation. Finally, these requirements and the related security architecture have been validated by means of a test-case that is based on a real home telehealth service in order to ensure their consistency, completeness, realism and verifiability.

## 1. Introduction

Telehealth aims to increase the efficiency of current healthcare services while it provides a basis for the provision of new services that improve quality of life [1]. However, telehealth services must overcome a number of challenges related to both the interaction of users with highly complex technologies and the compliance of these services with the standards of society [2]. Moreover, telehealth services are impacted by two key issues involved in the study of the relationship of people with technology [3,4]. First, users need to use the services implemented using these technologies because their health and quality of life depend on it; second, technical and technological skills among these users could be weak and impede their ability to interact with these types of complex services.

**Figure 1:**
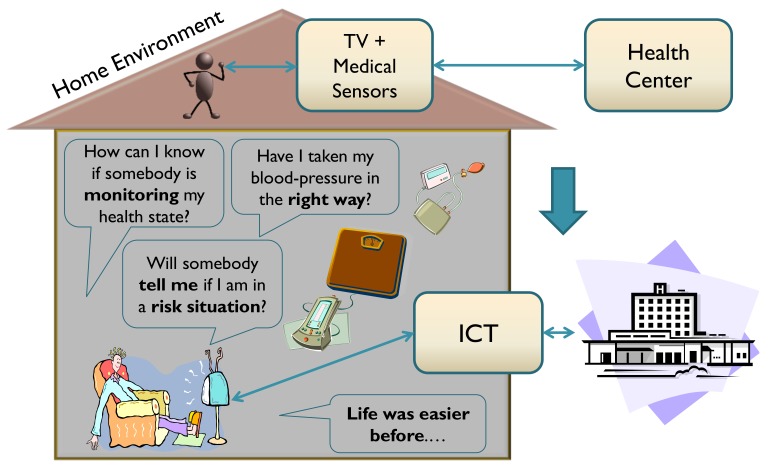
Generic representation of some user fears related to inclusion and deployment of telehealth services in the digital home [5,6].

Figure 1 depicts some of the typical concerns of a user of a telehealth service that is used from home (a remote monitoring service) that go unaddressed. Although transmission using Information and Communication Technologies (ICTs) and management of data using a health center may be suitable from the point of view of security, a number of fears may arise due to a lack of face-to-face contact with clinical personnel. In the absence of information and direct support from health personnel, patients may doubt that their data are being handled properly, that their therapy is being used correctly, or that they will receive an alert in the event of danger.

Furthermore, users of a telehealth service, as well as practically any other ICT-based service, usually lack the means to verify, as per the rules applicable in a given location, whether a transaction has been completed successfully. This leads to a lack of security in the event that conflicts occur during a transaction, as only the service provider possesses the telematics evidence, and this evidence is usually not secure against alteration [5]. These related to the security of information and patient privacy are especially critical for the promotion of the viability and acceptance of these services by society. 

There are numerous studies, recommendations and rules pertaining to matters of security in services and systems that manage healthcare [7,8,9,10]. However, key R&D work and regulations [11,12,13] have focused on the transmission and management of information by entities charged with the task of processing and storing this information. The common situation of disregarding users as active decision-makers within these systems is related to the fact that they are seen as the weakest link in the security chain [14,15], and this naturally hinders acceptance by intended users of services [16]. Unfortunately, the fact remains that the majority of services based on new technologies have not been designed to properly manage the fears of users [17,18].

This paper provides a comparative analysis of the security requirements that must be satisfied by telehealth services in order to properly manage user fears and expectations. The verification of the proposed requirements is focused on home telehealth services, as these are the type that are most often deployed. Thus, this study has yielded a series of semi-formal requirements, set forth below, that define the minimum features that a home-based telehealth service should have in order to fulfill the expectations of user-oriented security, which takes into account the personal, legal and regulatory aspects identified by the authors of this paper. The resulting set of requirements obtained from this systematic research is translated into the design of a security architecture centered on the user and mainly oriented towards the management of the concerns of users of home-based telehealth services. To verify the viability of this architecture and ensure the tacit validation of the underlying requirements, a mature home-based telenephrology system has been used [19,20,21]. In addition, this architecture has been used as a basis for security management for a case described in a previously reported study [22].

To facilitate understanding, the paper is organized as follows. After this introduction, the concept of User-Centered security is described in Section 2. Section 3 presents a systematic study of laws, regulations and security ethics codes that are relevant to healthcare environments in order to inform the conceptualization of User-Centered security requirements for telehealth services that is described in Section 4. Section 5 depicts the formalization of this theoretical design framework by defining a security software architecture that could be used within a real-world home telehealth system. Subsequently, the functionality of this architecture in terms of the security concerns under discussion is verified in Section 6. Finally, the discussion and conclusions reached by this study are presented in Section 7.

## 2. User-Centered Security

Security is a broad concept that is difficult to analyze and therefore clearly allows for several non-exclusive interpretations. Pau, in his dissertation [5], performed a systematic analysis of different security concepts that established the approach adopted by the authors of this article. This section summarizes this approach.

### 2.1. Need for More Accessible and Usable Security

Human interactions that are considered to be fair within a society are subject to certain norms, whether explicit or not, that give them a status of "sufficiently secure". Via these rules, the entities involved, whether they be individuals or other entities, acquire commitments and rights to maintain a certain status. Developed societies even have mechanisms of tutelage that are usually articulated in the form of laws and regulations. 

While ICT-based services must improve and/or discover new interactions that will enable the advancement of society by ensuring the rights of their members, they must also contribute to increasing their level of security. However, to attain this objective, telematics services must overcome, at minimum, the following goals:

- Providing value-based security that supports the achievement of the expected benefits for society in a fair way for all members [23].
- Satisfying the confidence needs of users related to the use of these services by offering perceptible and understandable guarantees of function [22].

In this sense, addressing these challenges incorrectly could lead to a situation where a telehealth service, which is potentially useful and necessary for users, is not allowed to operate if a sufficient level of confidence is not achieved in its operation or if it will entail a diminishment of any rights such as the right to privacy or the right to control one’s own image. In addition, it must be taken into account that this type of service is usually deployed within intimate user environments, such as the home. This generates a level of demand that is higher than that in other contexts in terms of rights to privacy and intimacy [22]. Hence, it is important that these types of sensitive services, in addition to performing their tasks in a secure and private manner, encourage the perception of security for its users. This explains why security concerns constitute one of the main impediments to the adoption of ICT technologies or other services provided through them [24,25,26].

To ensure acceptance by users of the security solutions offered by ICT services, these solutions must be included during the initial stages of telehealth service development instead of being mere adaptations of existing security mechanisms [27]. Thus, the services will be created based on security criteria that can be understood and managed by users and that are also compatible, without any ambiguity, with user rights. Therefore, this requires that user-oriented security principles be included in the development of telehealth services.

### 2.2. Evolution of User-Centered Security

The first definition of user-oriented security given in 1996 by Zurko described it as being composed of “security models, mechanisms, systems, and software that have usability as a primary motivation or goal” [28]. During the same time in which this concept was defined, a series of studies was undertaken to evaluate the usability of existing security mechanisms in ICT services [29,30]. These security usability studies have continued, uninterrupted, into the present [7,31,32,33].

However, several years after the introduction of the concept of user-oriented security, a change in focus emerged [34]. Previously, a majority of efforts had sought to evaluate and enhance existing mechanisms by lending them greater usability. In contrast, some authors began to argue for the need to redesign security mechanisms from the ground up and integrate them into an application as a functional component and not as an element to be added afterwards [35]. This approach attempted to avoid the conception of the functionality of the service and the security mechanisms separately. Thus, when functionality is designed, secure behavior is inherent and natural to the service or system and, in collaboration with the user, is able to reflect existing social interactions. The need to involve the user in security functions became even more apparent with the arrival of Ubiquitous Computing and the development of pervasive sensitive services [36,37].

Since 2003, a critical trend has developed related to traditional ideas regarding security and acceptance models [38]. This new trend has attempted to sensitize researchers and developers of sensitive services to the need of giving final users a type of control they are capable of understanding and thus prepare them for the dynamic and ubiquitous environments of the future [6,39,40]. The need to involve users in the evolution of systems stemming from the advance of technology is closely related to the increasing tendency to orient services towards the user. Because ICT services have become more present in users’ social interactions, they must be governed by the same conventions and social rules as other services in society. This implies an obligation on the part of ICT services to comply with established ethical and legal requirements to prevent users from being left helpless in situations arising from the use of these services.

### 2.3. Redefining User-Centered Security

Considering all of the new nuances that play a role in User-Centered Security, the definition given by Zurko in [28,41] may be insufficient. In addition to maintaining a high degree of usability, it is desirable for security mechanisms to be adequate in supporting the user during the use of services. Hence, in addition to offering an understandable, efficient and usable authentication system to the user, these mechanisms must also be adequate for all the services that the user requires, provide guarantees in terms of system operation and other users, and be compatible with prevailing laws, norms and ethical standards. Thus, the design of a system that includes User-Centered Security must be implemented not only using broadly tested mechanisms to ensure acceptance among users but must also be developed, from the beginning, in conformity with existing laws and regulations. This is the approach that is set forth in this paper.

## 3. Analysis of User-Centered Security in Telehealth Systems

One of the main objectives of this study is to introduce security restrictions in e-health systems in general, and telehealth services in particular, that will allow users to be aware of the secure nature of the transaction being made. These restrictions must coexist with those currently imposed by health centers and complement them. The goals, therefore, are to improve traditional security models that have already been implemented, strengthen global security and, possibly, ensure the psychological acceptance of patients. Due to the strong dependence of health services on legal regulations, our analysis focuses on the Spanish health system and takes into account pertinent legislation. This health system, which is compatible with European Union regulations, is sufficiently restrictive to be considered a significant case, and its analysis can be extended to other countries.

### 3.1. Ethical and Legal Facets

The analysis of the ethical and legal aspects that was performed at this point used, as a basis, the Guide to Information Security in Health Care [42]. The main purpose of this guide is as follows: “To assist health care professionals and managers in their task of safeguarding citizens’ rights and to foster a view of ICT as one of the essential components for protecting information.”

To meet these goals, the report covers the ethical, legal, organizational and technical facets of information security in health care. The ethical study in the report is of great interest for the attainment of security requirements. The concept of ethics is treated as a set of principles that orient the conduct of health professionals that are committed to providing patients with quality care. Ideally, ethics and law should go hand in hand, and this occurs with great efficiency in health care. However, there are certain matters that are difficult for the law to resolve, and others in which professional ethics take on special importance in terms of safeguarding patients’ rights.

The fundamental component of a patient’s clinical information is the electronic clinical documentation, also known as the electronic medical record, the electronic health record or the electronic personal health record. A clinical document, according to [42], *“expresses the reality of the patient throughout a timeframe and includes physical, mental and social aspects.”* At a technical level, it includes *“all information generated in relation to the doctor and other professionals in all medical acts.”* The report provides a verbatim quote from the Spanish Commission of Professional Ethics when it states, in [43] (in Spanish): *“the goods and values related to a clinical document are of extraordinary importance, as they are directly related to an individual’s fundamental rights, such as the right to privacy, to physical integrity, health, freedom and confidentiality.”*

The section on ethics in the report contains an important statement: “The purpose of the clinical document—to facilitate the medical care of the patient and record it [44] (in Spanish)—determines the professional ethics which govern it and condition access to this record.” It is important to note that, although the priority use of a clinical history is to facilitate both medical acts and the relationship between health professionals and the patient, other uses are also conceivable, including those related to the administration of justice, insurance claims, academic matters, and disease research. In any case, the clinical document contains information that is extraordinarily difficult to manage.

#### 3.1.1. Reliability

The reliability of health information is a right guaranteed by law for patients in most countries and a duty for professionals who must handle or store it. Although the law adequately protects these rights, professionals must be responsible in their use of information due to the vulnerability of patients, even when lodging a claim in defense of their rights. There are, nevertheless, a number of situations in which healthcare stakeholders are obligated to disclose a patient’s confidential data. Ultimately, professionals must take care to divulge only the information necessary to the proper persons for reasons of health.

#### 3.1.2. Availability and Treatment

Another important issue is the availability of data and it treatment. For patients, access to information is a legally recognized right in many countries, among them Spain [11,12,13]. But not only do patients have a right of access to their own clinical documents, their legal representatives or duly authorized family members may also be entitled to have access.

For their part, professionals must be able to share information from clinical documents in order to make a diagnosis or provide clinical treatment. This must be specified a priori via the explicit consent of the patient to guarantee his or her integrity. Secure official channels must be used to share this information and informal methods must, out of respect for the patient, be completed avoided. 

Of course, the obligations and rights established in the consent or contract for treatment may be limited, through legislative measures, as long as the situation requires. These kinds of special situations are addressed in several international regulations, such as Article 23 of the EU General Data Protection Regulation (GDPR) [11]. In this regard, it is worth remembering that it is possible for people who have custody of a patient’s clinical information to transfer the data due to public health or legal regulations for purposes that include avoiding damage to third parties, injunctions, or requirements related to birth or death certificates.

#### 3.1.3. Information Security

With respect to information security—which encompasses general confidentiality, integrity and conservation—a previous report [42] states: “From the point of view of ethics, the protection of clinical documents is entrusted to both professionals and health care systems.” The computerization of health care documents has led to the creation of the position of “Health Care Data Administrator.” This administrator must ensure the security, conservation and integrity of clinical documents. 

With regard to telemedicine, the Spanish Society for Health Care and Information Technology points out in [42] that neither distance nor the interposition of instruments may diminish the relationship of complete trust that must exist between a medical professional and the patient or weaken the interpersonal nature of that relationship. To prevent technology from becoming an obstacle and to ensure its compatibility with the enhancement of the quality of care provided to the patient without undermining the relevant values, the implications of incorporating technology must be analyzed both for users and for organizations that are adopting services. 

A previous report [20] (in Spanish) provides an in-depth description of the characteristics technology must offer in order for it to be used in healthcare. In its evaluation of medical technology, the report cites Van Wick [45], who identifies four essential features for the analysis of healthcare technologies:

- Technology is a phenomenon that must be created by people, as it does not arise on its own. This distinguishes it from natural phenomena, which are not the result of human creation.
- Technology is a means and is not an end in itself. This distinguishes it from art or other intellectual creations.
- Technology resides in entities composed of devices that include hardware and software and that also rely on human abilities. These entities may be useful for the study of technology.
- While the definition of technology includes human abilities as they relate to these entities, it does not involve the human being itself.

At a more technical level, the report [20] indicated the types of information included in clinical documents set several challenges and identified weak areas in the management of these records. Challenges include the treatment of clinical documents as a form of shared information in a way that can safeguard the legal and moral rights of all involved. 

Legal issues, which are basic to systems in which users may be at a clear disadvantage, are also addressed in [42]. The report states that, given the present importance of matters related to security, standards for good practices in terms of information security have been developed, such as International Organization for Standardization (ISO) 17799:2005. This standard is designed to facilitate compliance with the law and the implementation of information security in systems where it is applicable. Out of all the recommendations given in the report, the following are of particular relevance to this study:

- Identification of applicable laws.
- Protection of personal data.
- Prevention of the abuse of information systems.
- Use of cryptographic controls, including electronic signatures and the encryption of information.

### 3.2. Security and Human Factors

Petersen discusses in [46] mutual influences on security and human factors. An analysis was conducted that evaluated patient needs in relation to eHealth systems, bearing in mind that these systems are in different locations and that the people using them may have serious difficulties in understanding the reliability of the technological process, rising several fears that affect their acceptation of the solution. A special emphasis is placed on the security-related aspects, which are essential for providing confidence.

Among the possible difficulties encountered by users of eHealth systems, [46] points out the following:

- Management of sensitive data: the use of information that can result in rejection if the required trust is absent.
- Adaptation of systems for professionals: systems are created to meet the needs of professionals rather than patients.
- Understanding of difficulties by patients and stakeholders: such concepts are difficult to grasp for the majority of users.
- Integration with other eHealth systems and derived Telehealth services: integration of multiple systems often implies multiple functionalities that must be learned by patients and stakeholders.
- Use of special processes for initial configuration: most systems are designed to operate in a stable manner, but few are designed to provide adequate usability during their initial installation.

Personalization is one of the major challenges and a possible solution to many of the problems found in eHealth systems. Personalization not only means adapting the configuration according to the type of user but also altering the concepts used by the system to enable users to understand them sufficiently. Hence, the European Telecommunications Standards Institute (ETSI) has been working on defining a system of profiles applicable to ICT services. A previous report [47] includes a recommendation that defines the concept of user profile that is employed by the ETSI. This document provides a description of what a user profile is conceived to be, how it can help the user and some examples of its use. The ETSI has continued to develop the profile concept [48] and has defined the information that a user profile must include for the expression of preferences. This information includes not only applicable values and operations but also a language of rule definition that enables the specification of a new functionality in the profile, such as the *automatic modification of profiles according to the device or context*. In [49], the ETSI once again proposed an architectonic framework that could be used to support the personalization and management of user profiles in a manner that is consistent with [48]. The definition of the architectonic framework includes network requirements and processes in addition to aspects of privacy and security. The functionalities envisaged in the architecture include the management of the lifecycle of profile data, storage, synchronization, backup copies and access control. Of special interest are the security requirements imposed on the management of user profiles and profile storage. Both will be addressed in the definition of the security requirements included in the architecture of the telenephrology system.

In continuance of its work on personalization using profiles, ETSI also recently presented a very early draft that contextualizes all issues related to user profiles in eHealth systems [50]. Owing to the as-yet undeveloped status of the draft, no clear conclusions can be drawn based on the proposals, as the document itself states that further in-depth study is required, particularly in regard to security. However, the draft does indicate that issues related to privacy and the provision of user assurances will adhere to the same principles as the user experience guide for telecare services [50], a document that is fundamental to facilitating acceptance of ICT services by final users.

### 3.3. Standardizing Security in Telehealth Services

The present standards for health information systems reflect security considerations that must be taken into account. Healthcare systems include many organizations that are focused on the generation of standards for the introduction of new technology. Quite often, these organizations develop standards that are incompatible with other standards, and often will define, in a manner that is similar but not reusable, recommendations for the same functionalities. To expedite this inefficient process, a working group known as the eHealth Standardization and Coordination Group (eHSCG) was created in 2003. The eHSCG presently has members as influential as the World Health Organization (WHO), the Technical Committee of the ISO, which is charged with ensuring the compatibility of independent eHealth applications (ISO TC/215.), the Health Level 7 (HL7) and the Technical Committee of the CEN, which is charged with standardizing systems related to electronic medical records, knowledge representation, and messaging (CEN/TC 251.).

The objective of the eHSCG is to promote cooperation between the leading entities in the field of eHealth. The starting point of its work is the voluntary acceptance of all entities, as it has no legal authority. The ITU Telecommunication Standardization Sector (ITU-T) released a technical document on telemedicine in 2006 [51]. This document addresses security in telemedicine and telehealth and is divided into two parts: the first part addresses security in telecommunications and information technology in general using generic and well-known concepts of security, while the second part concerns the information managed by health care systems. Specifically, it discusses issues of privacy and confidentiality of information. Thus, it offers a review of the different agencies engaged in the standardization of security (many of which have been cited previously as members of the eHSCG) and identifies existing directions in the development of the security field. The report states that the main lines of development relate to the security of the exchange of data, access of informational records by patients and professionals and access control with the possibility of audits. The conclusions in the section on security make it clear that the integration of different specifications, solutions and standards presented the greatest challenge as of the date of the report.

Finally, the Personal Connected Health Alliance (PCHAlliance), a non-profit organization formed by HIMSS, presented in 2008 a guidelines for ensuring the interoperability of health devices and Electronic Health Records through specific secure networks, the Continua Design Guidelines (CDG) [52]. This document points out a secure end-to-end ICT framework for personal connected health and care using open standards, to create a secure and interoperable health data exchange in personal connected health. Nowadays, PCHAlliance maintains this framework with the last version of the document [53] where they are expanded its capabilities to support cloud computing, HL7-FHIR, end-to-end security and new device specifications, among other features related with interoperability.

## 4. Conceptualization of User-Oriented Security for Telehealth Services

The analysis of the context and needs that have been previously described, as well as the knowledge acquired by authors of previous reports [22], have been conceptualized in the form of semi-formal requirements. These requirements define a design framework for telehealth services that has the objective of maximizing the perception of security and privacy by users. These requirements are set forth below:

R1. The aim of telehealth is to generalize healthcare services to make them more accessible and affordable to citizens, regardless of their spatiotemporal or socio-economic situation [54,55,56]. Existing security conditions must not alter or limit this objective.
R2. The system must ensure custody over all information related to the medical issues of the patient, independently of their origin or source [29,31,42]. This information will be stored in an electronic clinical document [57,58]. Moreover, this information, which will increase throughout the patient’s life, must remain integrated to enable the system to access it in a unique manner; however, it must also be logically segmented for the purposes of access restrictions.
R3. Certain data in clinical documents may be disclosed in legally mandated situations, such as in response to court orders, express authorization that is provided by the patient to certain users, or in circumstances of public risk [11,12]. All data disclosed will be recorded as will the circumstance in which it occurred, how the patient can track it and check it at any time.
R4. The communication between the patient and the health professional must follow the criteria of confidentiality, authentication, non-repudiation and integrity [51].
R5. The patient’s data, after an adequate process of loss of traceability (provision of anonymity), can be used for research purposes. To do this, the ethics committee or the Data Protection Officer, if exist, of each entity will make a final decision in this regard.
R6. The possibility of sharing data from a patient’s clinical documents with other healthcare centers or units should be offered in order to provide better overall treatment and care. This data sharing must follow certain rules and policies [11,12]. The sharing policy must provide the necessary mechanisms to allow audits by users or external legal entities.
R7. Patients have the right to be in possession of their clinical documentation (owner). In turn, they may authorize the processing of this information for a specific purpose, which is established a priori, in a way that safeguards the rights of the owner (patient). However, consideration should be given to limiting the scope of the consent obligations and the owner’s rights for certain special situations in a way that is covered and protected according to current legislation (GDPR: Article 23).
R8. Patients must be able to eliminate all information or traces related to him or her (GDPR: Article 17).
R9. The patient must be aware of what is occurring in their interactions with a doctor. The patient must also be able to expressly confirm the data exchange.
R10. Security hardware must be used as the basis for identification of the patient, and the public key infrastructure (PKI) must be used for the certification of credentials.
R11. Secure terminals must be used for generation of an electronic signature pursuant to Spanish law. The requirements of Spanish law are sufficiently generic and, at the same time, sufficiently demanding to comply with European laws and regulations.
R12. The security measures that are implemented must enable mechanisms of personalization that enhance the perception of security by the user and, consequently, favor usability.

## 5. Formalization of User-Centered Security in Telehealth Services

The conceptualization of User-Centered Security described in the previous section can be used as a structured solution to guide future implementations. This structured solution is based on two main concepts: a security reference architecture and an information exchange model. 

### 5.1. Security Reference Architecture

**Figure 2:**
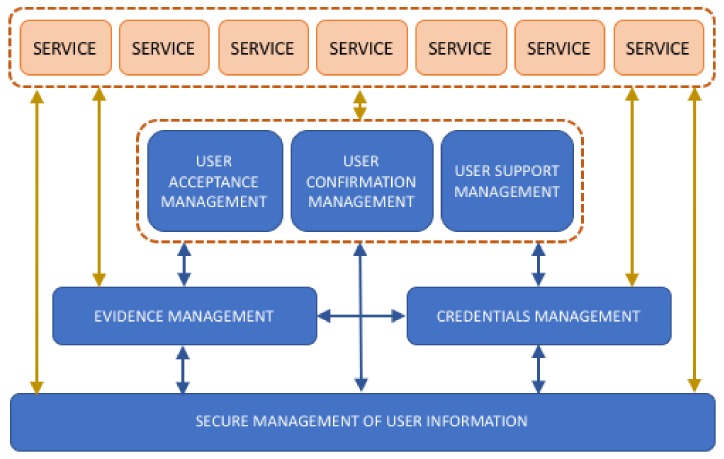
Graphic overview of a reference service-based architecture proposed as a formalization of the User-Centered security concept.

The reference architecture represents a type of template that meets all of the functional and operational requirements that are involved in the development of a specific type of system [59]. According to the guidelines given by the International Organization for Standardization (ISO), it has been assumed that the best basic reference model is service-based. Based on this, the presented reference architecture has translated the above requirements into a minimum and sufficient set of services [60]. Figure 2 depicts a service-based architecture that is based on the proposed User-Centered security concept.

Defining the requirements in the form of services will facilitate their identification and implementation, either as part of telehealth services or as independent and reusable elements in other contexts. Thus, this formalization is designed to be included in already completed systems in order to improve them based on the concepts of User-Centered security. The main functions of each of the proposed services are described in Table 1.

### 5.2. Information Exchange Model Based on the Contract-Document

The concept of the Contract-Document (C-D) is defined in [22] and has been used in this study as the basis of information exchange between entities and to offer a security and privacy guarantee to users.

In many applications, the semantics of the transaction are implicit in the exchange of messages without being reflected in the pieces of information that are held by each user. The information model based on the proposed C-D avoids this situation using the incremental creation of an electronic document that includes all the entities involved in a transaction. All actions are recorded in the document, with adequate security guarantees. This allows for knowledge of not only the final state of the transaction but also how and by whom it was performed.

**Figure 3:**
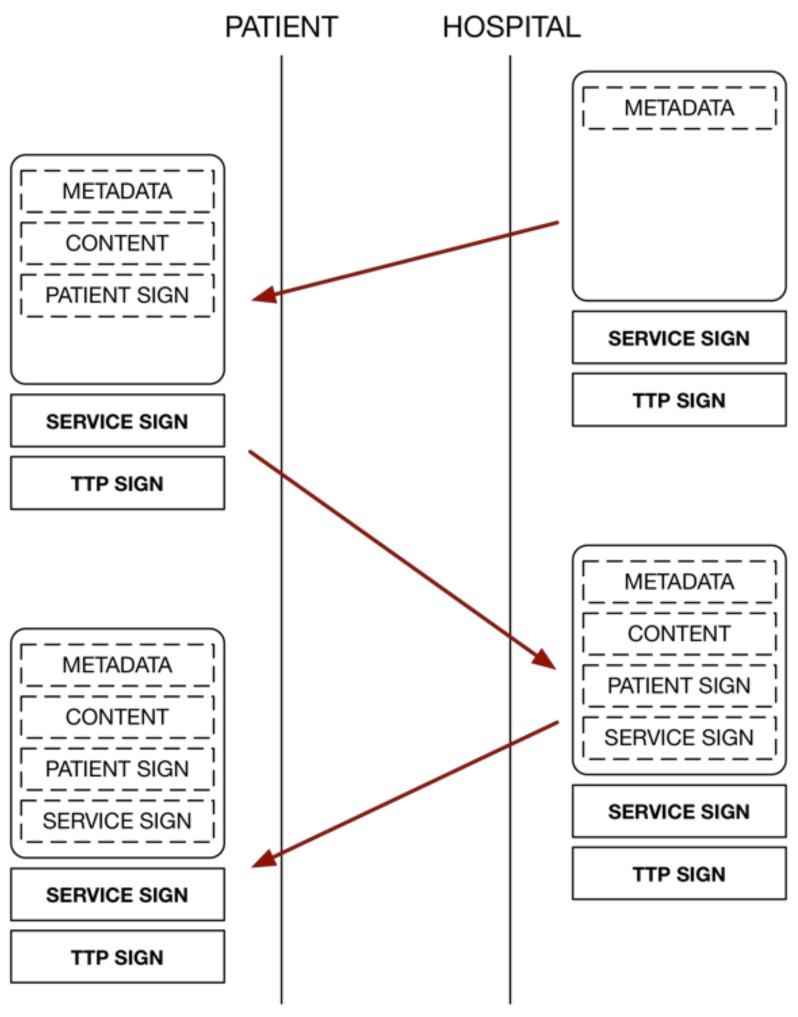
Information exchange model based on the concept of the Contract-Document.

As shown in Figure 3, at the end of a transaction, both the hospital and the patients will have a copy of the transaction that faithfully reflects, in an understandable manner, both the content of the transaction and the actions that were carried out by both parties. Using this approach, the interaction between the entities is controlled by the document itself via the content that must be provided by both entities. The design of the fields to be used in the document, as well as the protection given to each of the fields, depends on the application in question.

## 6. Case Study: A Confident Telenephrology System

The case study in which the proposed reference architecture was used is based on a telenephrology system that was first described in [19] and implemented in [21]. The objective of this telehealth service was to ensure that patients engage in telehealth sessions with healthcare personnel in which information about their state of health was transmitted. However, during the trials, certain fears as to whether services was being performed properly were voiced, especially if the session did not include a videoconference with health personnel. Based on this, the security components within the architecture were redesigned based on several of the User-Centered security ideas presented in this report to improve acceptance among users.

### 6.1. Overview of the Case Study

A telenephrology system is a special type of telemonitoring system that monitors and records the biomedical variables of patients with renal insufficiency. Patients treated in a hospital nephrological service, specifically patients with renal failure, can greatly benefit from constant monitoring of biomedical variables such as blood pressure or weight. Health specialists can examine the alterations and trends in these variables in order to detect anomalies occurring during the treatment of these patients. Therefore, it is necessary to transmit measurements from the home to the hospital in a way that is secure and understandable by patients, similarly to how this would occur at the hospital.

### 6.2. Security Module Used in the System under Study

In this system, described in [19], patients use measuring devices (scale and tensiometer), and when the measurement is finished, they transmit it to the data acquisition center (media center). In the media center, the measures are stored until all the necessary measures to be sent during that session are obtained. When the measurements are received, the specialist can provide confirmation to the patient via a teleconference system. The system will indicate to the patients, by way of the television to which the system is connected, the status of the measurements, and patients can even view a history of all the measurements taken. The information transmissions are sent via email using S/MIME encapsulation to encrypt the data.

**Figure 4:**
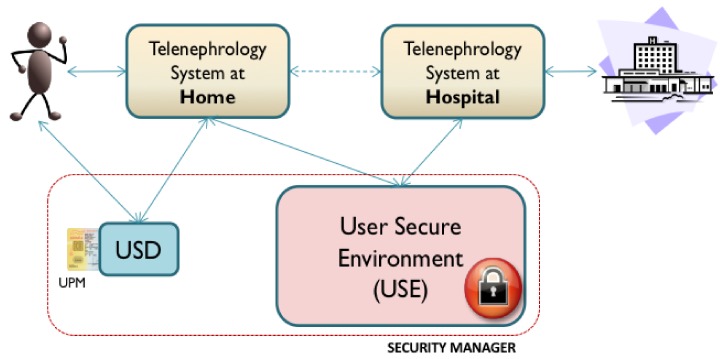
Overall graphic depiction of the components of the new security manager based on the User-Centered security architecture and the relationships between these and the telenephrology system.

As has been previously mentioned, in response to the lack of confidence described by the users of the system, it was proposed to update the security manager using the proposed User-Centered security architecture. This architecture proposes a number of functionalities that must be included; for this purpose, three new security elements have been defined for the telenephrology system: the UPM, the USD and the USE. These elements provide for both usable and secure cryptographic credential management and information storage focused on security for the user. These elements, combined with the principles used for the information exchange model based on the previously described C-D, allow for the coverage of all services defined in the reference security architecture, and therefore, all User-Centered security requirements that have been previously proposed. Figure 4 depicts the relationships of these elements within the telenephrology service. Each component is described in the following. 

#### 6.2.1. Telehealth System

The telehealth system continues to offer users (the patients and the hospital) the same services. The patients’ measurements are obtained and sent by e-mail (encapsulated by S/MIME) to the hospital. However, there are two other entities with which it interacts directly: the User Security Device (USD), through which the user can obtain information regarding the security of the transaction, and the User Secure Environment (USE), which stores and allows access to the C-Ds generated during transactions.

#### 6.2.2. User Security Device (USD)

Critical security interactions are performed using an independent device that is separate from the terminal from which the service was accessed. The aim of this device is to offer a suitable interface for users, prevent Man-In-The-Middle (MITM) problems and enable personalization of the User Presence Module (UPM). Currently, multiple possibilities are available for the implementation of this kind of device: smartphones, Smart Card readers, USB tokens, and many others. The selected technology for USD functionality is the Smart Card reader. 

#### 6.2.3. User Presence Module (UPM)

This module should be closely integrated with the USD. It implements personalization and secure storage of user credentials. The cryptographic credentials and available profiles for a user are stored on the device with guaranteed security. The USD communicates with this device to execute information exchange operations and transactions required by users and the C-D. Within the current architecture, this has been implemented on a Smart Card. 

#### 6.2.4. User Secure Environment (USE)

The USE is a secure repository of information for the user. The system stores, manages and retrieves all the security information produced by the telenephrology system during transactions in the form of a C-D. Users can store and securely retrieve this information at any time. Clinicians and other medical stakeholders can also retrieve user information if they have the express permission to do so. Thus, it is possible that the environment may provide clinical information from patients that have been previously anonymized for the purposes of research.

### 6.3. Description of the Operation of the System after Improving the Security Module

By including the User-Centered security module, which is implemented using the architecture described in this paper, the operation of the telenephrology system should be conducted as follows.

As noted previously, a basis of the new security model is the C-D. The hospital generates a C-D that is properly validated using traditional security methods, such as PKI, digital signatures or a unique reference number. The C-D will contain nothing within the section that is to be filled in by the patient or in other sections that are to be filled in by the final party. This template must first be in the possession of the patient (the process will be further discussed below). When the patient obtains measurements at home, the system fills in the C-D with the measurements and then sends the C-D to the USD. The USD verifies that the user has validated the measurements with a signature and gives the user, in a clear and unambiguous manner, the option to validate the document. If the user accepts, the USD will request a UPM signature and fill in the appropriate field in the C-D. 

Once the patient portion of the document has been filled in with the clinical measurements and the necessary security measures have been completed, it is sent via S/MIME to the hospital. It is also sent via S/MIME to the USE for storage for future use. The system at the hospital processes the S/MIME message, obtains the C-D and verifies that a single field remains to be filled in, which approval is provided for its receipt (again, with a signature). It fills in the field and sends the patient’s final C-D via S/MIME. Attached to the same message, a new blank C-D is sent with a new reference number for subsequent use. If the patient should lack a blank C-D to start a new transaction, one may be requested at any time using a specific S/MIME. The interface of the telenephrology system must enable this action. When the document is received by the telenephrology system, it is displayed on the interface and sent to the USD for verification. The USD will again provide the user with clear information that indicates that the process has been completed successfully and will ask the telenephrology system to send the new information to the USE. In the USE, the previously-acquired, incomplete information will be replaced with the new information (identified with a reference number).

### 6.4. Verification of Requirements

In order to begin the validation of the fulfillment of the requirements established in this study, Table 2 indicates which elements in the new architecture satisfy each User-Centered security requirement.

## 7. Discussion and Conclusions

Our analysis of regulations related to security in health services found no documents that explicitly addressed the relationship between ICT services and users in terms of user skills and expectations. The majority of security mechanisms have been created to be understood by users with a sufficient technical background. This reduces the final users’ trust in critical services such as telemedicine services. It also tends to reinforce the axiom that states that final users are the weakest link in the security chain, even though security has not been designed with users in mind despite the fact that they are the party that most requires protection.

The absence of multidisciplinary teams focused on the implementation of security has prevented the inclusion of security solutions that can be directly beneficial to users. In an effort to remedy this failing, this paper presents a User-Centered security concept and its characterization in the form of semiformal requirements. The viability of this characterization has been analyzed via its formalization in a security software architecture. This reference architecture, based on services, offers a design guide or template that can be used to implement the User-Centered Security concept during the development of telehealth systems or services. 

In terms of the validation of the requirements [60], these requirements do not offer a different description of the same functionality (consistency), but instead represent the minimum set of needs that are addressed (completeness) and can be implemented using the current resources (realism). Also, by means of a case study, it has been possible to analyze how that formalization can be applied to a real-world scenario and how every requirement that has been proposed is addressed by this new solution (verifiability). In this sense, this research proposes the use of a new Security Module/Manager for the implementation of the proposed reference architecture. 

In the context of a telenephrology system, this system tends to minimize the risks to which users are exposed by supporting specific security devices (UPM and USD) and the use of an external entity to protect users’ rights and interests (USE). This external entity also ensures that medical information is available to both the user who owns the information and external entities that must make use of it. External entities must adhere to a strict access policy that has been designed to safeguard user rights and that is known to users for accessing the information, such as the GDPR.

However, the inclusion of the proposed reference security architecture in a real telehealth service and a previously implemented telenephrology service has been possible but has led to a drastic change in the original design that involved the modification of not only the security module but most of the subsystems that were present. This strengthens the idea that security must be treated as a system functionality and should be included in the initial design of a telehealth service. For that reason, User-Centered security, its characterization and the proposed software architecture all offer to the stakeholder proper tools that can be used to address this problem in collaboration with final users. Thus, this study may help teams that are developing services to consider security issues during the earliest stages of a project. Both the process of analysis and the characterization of the User-Centered Security concept yielded insights, and their formalization within an implementable telematics architecture can be extended to areas outside of healthcare in which sensitive ICT services are managed.

Finally, it is necessary to analyze the compatibility of the characterization of the User-Centered Security concept presented with the GDPR. The GDPR is a European regulation that ensures the protection of natural persons regarding the processing of personal data and the rules regarding the free circulation of these data. In addition, when dealing with telehealth applications and services, we are faced with a special category of personal data, which are data concerning health. To deal with this issue, the GDPR defines the consent that must be obtained to manage this type of personal data [61], which must be explicit, informed and revocable (GDPR: Article 4 (11)). Thus, this consent establishes a type of contract between the natural person and the information processor, which facilitates their supervision and management under the current legislation.

In this sense, the C-D defined in this paper offers the possibility of establishing explicit consent between the interested parties and the establishment of an information register in which all the actions performed by the entities involved in a processing are reflected. Thus, the C-D allows an entity to know the status of information processing at any time, which facilitates monitoring by the natural person and owner of the information, as well as an audit if necessary. A future improvement of the C-D could include the integration with Blockchain technologies. Blockchain has several challenges when applied to telehealth systems [62]. The integration of the immutable capabilities of Blockchain, with the flexibility offered by the C-D, could address all the security requirements proposed in this paper in a suitable and reliable way. The other elements of the defined reference architecture provide a solution for ensuring security within this model of information exchange and empower the natural person before all other entities.

## Figures and Tables

**Table 1:** Description of the services that comprise the reference architecture.

Service	Name	Description
1	Secure management of user information	This is the location where the user can deposit all information considered critical from the point of view of security. The safe user space must protect information to safeguard the rights of the user.
2	Credentials management	This manages all the necessary credentials to guarantee the identity of the user and the services accessed. It must offer mechanisms for obtaining new credentials, updating them and classifying them according to their usefulness.
3	Evidence management	It gathers all the necessary functionalities to create a system of evidence that guarantees security for users. It will also provide adequate storage and facilitate the recovery of evidence.
4	User acceptance management	This service formalizes the mechanisms necessary to improve psychological acceptance of the user. To do this, the elements must be incorporated in a way that improves the understanding of the transaction by the user, increases confidence that the service is being provided properly, and ensures the continuity of the service at any time.
5	User Confirmation management	This encompasses all functionality required for user confirmation (user interaction) to be conducted in a secure environment. The user must perform this confirmation in a centralized manner and with secure devices.
6	User Support management	Using this service, the user may request help from third parties prior to a transaction.

**Table 2:** Analysis of which elements in the new security manager address each User-Centered security requirement that has been proposed.

Requirement	Elements Involved	Description
1	All	Security is not an aim in itself but should be customized to the final application. The C-D format must be specific to telenephrology.
2, 3, 5, 6, 7, 8	USE	The USE enables users to manage their own clinical documents. For a telenephrology system, this implies the management of medications and the entities to which they have been sent. Also, this enables the disclosure of data to third parties under previously agreed conditions.
4	C-D, S/MIME	The security services discussed are provided at two levels: first, the C-D must be designed to satisfy security requirements; and second, the S/MIME wrapping also provides several of the security services that are required.
9	C-D	By its very nature, the C-D describes all the events in a transaction.
10	UPM	The UPM is implemented using Smart Cards as a way to enhance the security and usability of the system for users.
11	UPM and USD	Devices responsible for interacting with the user must be reliable and trusted. Both the UPM and the USD must be constructed according to established computing principles.
12	UPM and USD	Using the personalization information in the UPM, the USD adapts to the patient’s capacity in terms of accessibility and comprehension.
